# The first consecutive 5000 patients with Coronavirus Disease 2019 from Qatar; a nation-wide cohort study

**DOI:** 10.1186/s12879-020-05511-8

**Published:** 2020-10-19

**Authors:** Ali S. Omrani, Muna A. Almaslamani, Joanne Daghfal, Rand A. Alattar, Mohamed Elgara, Shahd H. Shaar, Tawheeda B. H. Ibrahim, Ahmed Zaqout, Dana Bakdach, Abdelrauof M. Akkari, Anas Baiou, Bassem Alhariri, Reem Elajez, Ahmed A. M. Husain, Mohamed N. Badawi, Fatma Ben Abid, Sulieman H. Abu Jarir, Shiema Abdalla, Anvar Kaleeckal, Kris Choda, Venkateswara R. Chinta, Mohamed A. Sherbash, Khalil Al-Ismail, Mohammed Abukhattab, Ali Ait Hssain, Peter V. Coyle, Roberto Bertollini, Michael P. Frenneaux, Abdullatif Alkhal, Hanan M. Al-Kuwari

**Affiliations:** 1 Communicable Diseases Center, Hamad Medical Corporation, Doha, Qatar PO Box 3050,; 2 Medical Education Center, Hamad Medical Corporation, Doha, Qatar PO Box 3050,; 3 Division of Critical Care, Department of Medicine, Hamad Medical Corporation, Doha, Qatar PO Box 3050,; 4 Hazm Mebaireek General Hospital, Hamad Medical Corporation, Doha, Qatar; 5 Hamad General Hospital, Hamad Medical Corporation, Doha, Qatar PO Box 3050,; 6 Business Intelligence Unit, Hamad Medical Corporation, Doha, Qatar PO Box 3050,; 7 Rumailah Hospital, Hamad Medical Corporation, Doha, Qatar PO Box 3050,; 8 Division of Virology, Hamad Medical Corporation, Doha, Qatar PO Box 3050,; 9 Ministry of Public Health, Doha, Qatar PO Box 42,; 10 Scientific, Academic and Faculty Affairs, Hamad Medical Corporation, Doha, Qatar PO Box 3050,

**Keywords:** Coronavirus, COVID-19, SARS-CoV-2, Mortality, Qatar

## Abstract

**Background:**

There are limited data on Coronavirus Disease 2019 (COVID-19) outcomes at a national level, and none after 60 days of follow up. The aim of this study was to describe national, 60-day all-cause mortality associated with COVID-19, and to identify risk factors associated with admission to an intensive care unit (ICU).

**Methods:**

This was a retrospective cohort study including the first consecutive 5000 patients with COVID-19 in Qatar who completed 60 days of follow up by June 17, 2020. The primary outcome was all-cause mortality at 60 days after COVID-19 diagnosis. In addition, we explored risk factors for admission to ICU.

**Results:**

Included patients were diagnosed with COVID-19 between February 28 and April 17, 2020. The majority (4436, 88.7%) were males and the median age was 35 years [interquartile range (IQR) 28–43]. By 60 days after COVID-19 diagnosis, 14 patients (0.28%) had died, 10 (0.2%) were still in hospital, and two (0.04%) were still in ICU. Fatal COVID-19 cases had a median age of 59.5 years (IQR 55.8–68), and were mostly males (13, 92.9%). All included pregnant women (26, 0.5%), children (131, 2.6%), and healthcare workers (135, 2.7%) were alive and not hospitalized at the end of follow up.

A total of 1424 patients (28.5%) required hospitalization, out of which 108 (7.6%) were admitted to ICU. Most frequent co-morbidities in hospitalized adults were diabetes (23.2%), and hypertension (20.7%). Multivariable logistic regression showed that older age [adjusted odds ratio (aOR) 1.041, 95% confidence interval (CI) 1.022–1.061 per year increase; *P* < 0.001], male sex (aOR 4.375, 95% CI 1.964–9.744; *P* < 0.001), diabetes (aOR 1.698, 95% CI 1.050–2.746; P 0.031), chronic kidney disease (aOR 3.590, 95% CI 1.596–8.079, P 0.002), and higher BMI (aOR 1.067, 95% CI 1.027–1.108 per unit increase; P 0.001), were all independently associated with increased risk of ICU admission.

**Conclusions:**

In a relatively younger national cohort with a low co-morbidity burden, COVID-19 was associated with low all-cause mortality. Independent risk factors for ICU admission included older age, male sex, higher BMI, and co-existing diabetes or chronic kidney disease.

**Supplementary information:**

**Supplementary information** accompanies this paper at 10.1186/s12879-020-05511-8.

## Background

Severe Acute Respiratory Syndrome Coronavirus 2 (SARS-CoV-2), the cause of Coronavirus Disease 2019 (COVID-19), emerged in China in late 2019. By July 12, 2020, more than 12 million confirmed SARS-CoV-2 infections were confirmed worldwide, with over 500 thousand associated deaths [1].

Based on the number of deaths as a proportion of reported COVID-19 cases, the overall estimated COVID-19-associated mortality rate is around 5.7% [2]. However, the accuracy of such a figure is uncertain given the variation in case finding policies from one healthcare setting to another [3, 4]. Furthermore, reported mortality has been mostly based on in-hospital outcomes or relatively short follow up [5–9]. In their recently published recommendations for a minimal common outcome measure set for COVID-19 research, the World Health Organization (WHO) favored that mortality outcomes are assessed at 60 days [10].

Single and multi-center cohort studies suggested that risk factors for severe COVID-19 include male sex, older age, and the presence of multiple comorbidities [7, 9, 11]. The extent to which such risk factors are important at a population level in settings with ample healthcare resources, a COVID-19 control program based on active case finding and isolation, and a low burden of comorbidities, is unknown.

In this study, we describe 60-day outcomes of a nationwide COVID-19 cohort from Qatar, and explore patient characteristics associated with the need for admission to an intensive care unit (ICU).

## Methods

### Study setting

Hamad Medical Corporation (HMC) encompasses multiple hospital facilities and provides all COVID-19 medical care for the 2.8 million population of Qatar. In response to the COVID-19 pandemic, existing clinical services were re-organized and two brand new hospital facilities were opened ahead of their originally planned dates. In total, non-ICU bed capacity was increased from 2143 to 3469 (61.9% increase), and ICU beds from 130 to 529 (306.9% increase). From a healthcare delivery perspective, HMC defines adults as those aged above 14 years.

SARS-CoV-2 infection was diagnosed by real-time polymerase chain reaction (RT-PCR) assays TaqPath COVID-19 Combo Kit (Thermo Fisher Scientific, Waltham, Massachusetts) or Cobas SARS-CoV-2 Test (Roche Diagnostics, Rotkreuz, Switzerland) on respiratory tract specimens. Severity of COVID-19 was classified according to the WHO guidelines [11]. SARS-CoV-2 testing was offered to all individuals presenting with symptoms suggestive of COVID-19, known close contacts of confirmed cases including healthcare workers, and all returning travelers.

Patients with asymptomatic SARS-CoV-2 infection or mild COVID-19 without significant co-morbidities were isolated in dedicated community facilities until they had two consecutive negative SARS-CoV-2 RT-PCR results from upper airway samples taken more than 24 h apart. COVID-19 patients with significant co-morbidities or moderate to severe disease were hospitalized for inpatient management. Standard care for hospitalized patients involved supportive care and investigational antiviral therapy. Individual regimens were selected by the treating physicians based on severity of disease, the presence of contra-indications or potential drug-drug interactions, and the patients’ preferences. Twenty five individuals included in this study had been elsewhere reported [12].

### Procedures

We used the HMC COVID-19 database to identify the first consecutive 5000 patients with RT-PCR-confirmed COVID-19 who would complete 60 days of follow up from date of diagnosis by June 17, 2020. During the period between May 24 and June 18, 2020, clinical and laboratory data were retrieved from HMC’s electronic healthcare system. Final status 60 days after COVID-19 diagnosis was ascertained against the electronic healthcare system and Qatar’s national deaths records.

The report was prepared according the Strengthening the Reporting of Observational Studies in Epidemiology (STROBE) recommendations [13].

### Outcomes

The primary endpoint was all-cause mortality within 60 days after RT-PCR confirmation of SARS-CoV-2 infection. For hospitalized patients, we also assessed risk factors for admission to ICU.

### Statistical analysis

We summarized categorical data as numbers and percentages and compared them using Pearson’s chi-squared or Fisher’s exact test, as appropriate. Continuous data are presented as medians and interquartile ranges (IQR) and compared among groups using Wilcoxon rank-sum test. The majority (82 patients, 75.2%) of admissions to ICU occurred within of the first 48 h from hospitalization. We therefore used logistic regression to explore predictors of admission to ICU.

Baseline variables were included in the univariable logistic regression analysis if their between groups differences were associated with *P* values of < 0.05. Independent variables in the multivariable regression model were chosen based on their association with *P* values of < 0.1 in the univariable logistic regression, and on their ready availability before any COVID-19-related clinical evaluation. Due to the number of events in the study, we limited the number of independent variables in the multivariable regression analysis to eight to avoid overfitting the model. The final multivariable logistic regression model included age, male sex, body mass index (BMI), defined as body weight in kilograms divided by squared height in meters, and co-existing diabetes mellitus, systemic hypertension, coronary artery disease, chronic liver disease, and chronic kidney disease. Multiple imputations approach was applied for variables with > 5% missingness.

All *P* values were two-sided with a threshold of < 0.05 for statistical significance. Statistical analyses were performed using Stata Statistical Software Release 15.1 (StataCorp LLC, College Station, Texas).

## Results

**Fig. 1:**
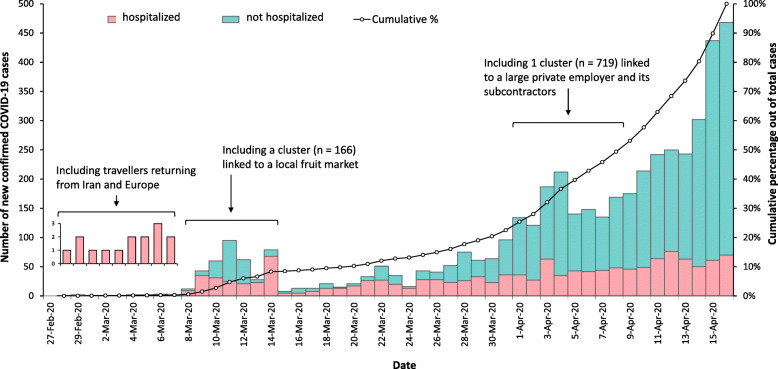
Epidemic Curve of the Cases of Coronavirus Disease 2019 (COVID-19) in Qatar. Daily numbers (Y axis) and cumulative count (Z axis) of confirmed cases are plotted by date (X axis) samples were taken. Inserts describe significant events with their corresponding dates. A total of 474 cases were diagnosed on April 17, 2020, of which 251 are included in this report

**Fig. 2:**
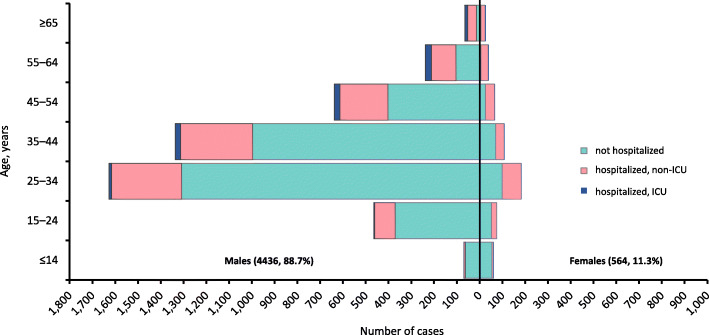
Patients with Coronavirus Disease 2019 (COVID-19) Stratified by Age Group and Sex. X axis, numbers by sex; Y axis age group (years)

Individuals included in this study were diagnosed with SARS-CoV-2 infection between February 28 and April 17, 2020. Initial SARS-CoV-2 cases were diagnosed in travelers returning from Iran and Europe. Sustained local transmission became established thereafter (Fig. 1). Of the 5000 RT-PCR-confirmed COVID-19 cases included in this report, 4436 (88.7%) were in males and the majority belonged to age groups 25–34 years (1811, 36.2%) and 35–44 years (1445, 28.9%) (Fig. 2). The cohort included 131 (2.6%) individuals aged 14 years or less, 26 (0.5%) pregnant women and 135 (2.7%) healthcare workers (Table S1, appendix).

**Fig. 3:**
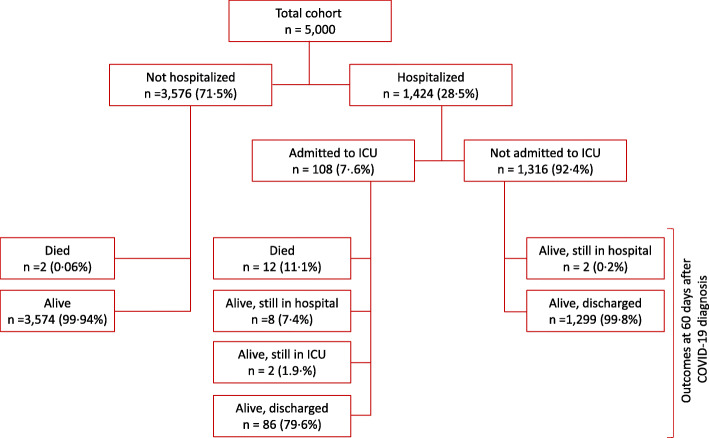
60-Day Outcomes of Patients with Coronavirus Disease 2019 (COVID-19)

Most individuals in this study did not require hospitalization (3576, 71.5%). Those who were not hospitalized were significantly younger and had fewer co-existing chronic medical conditions (Table S1, appendix). Of 1424 patients who required hospitalization, 108 (7.6%) were admitted to ICU. Overall, 60 days after COVID-19 diagnosis, 14 patients (0.28%) had died, 10 patients (0.2%) were still in hospital and two (0.04%) were still in ICU (Fig. 3).

### Hospitalized adults

Out of 4869 individuals aged > 14 included in this report, 1409 (28.9%) were hospitalized. The majority (1167, 82.8%) were males and the median age was 39 years (IQR 30–50). Nationalities from WHO’s South-East Asia Region (791, 56.1%) and Eastern Mediterranean Region (485, 34.4%) were most frequent. Diabetes (327, 23.2%) and hypertension (292, 20.7%) were the most common co-existing medical conditions. Fever (58.3%) and cough (59.1%) were the most common presenting symptoms. Median BMI was 26.8 kg/m^2^ (IQR 23.9–29.8). Most patients (1233, 87.5%) did not require oxygen therapy within the first 24 h of hospitalization. Hydroxychloroquine (1044, 74.1%), azithromycin (898, 63.7%), and lopinavir-ritonavir (522, 37%) were the most commonly used investigational antiviral agents.

Compared with those who did not require ICU admission, ICU patients were significantly more likely to be males (P 0.005), have higher median age (*P* < 0.001) and to have multiple co-morbidities (*P* < 0.001) (Table 1). They also had significantly higher median BMI (28.2 versus 26.6, *P* < 0.001) and were more likely to present with fever, cough and dyspnea (*P* < 0.001 for each). Within the first 24 h of hospitalization, ICU patients had significantly higher median heart rate (95 versus 86 per minute, *P* < 0.001), and respiratory rate (26.5 versus 19 per minute, *P* < 0.001), significantly lower oxygen saturation (94% versus 98%, *P* < 0.001) (Table 1). In addition, baseline blood investigations from ICU patient were significantly more likely to show lower median peripheral lymphocyte count (1.0 versus 1.7 ×  10^9^/L, *P* < 0.001), and higher median serum creatinine (90 versus 80 μmol/L, *P* < 0.001), and C-reactive protein (CRP) (107.7 versus 7, *P* < 0.001). Complications such as acute kidney injury (43.5% versus 1.5%), and myocardial injury (14.8% versus 0.3%, *P* < 0.001) were more common in ICU compared with non-ICU patients. Other baseline characteristics, management, and complications variables in hospitalized COVID-19 adults included in this study are shown in Table 1.

**Table 1:** Baseline characteristics, management and complications in adults hospitalized with Coronavirus Disease 2019 in Qatar

Variable	Non-ICU (***n*** = 1301)	ICU (***n*** = 108)	***P*** value
**Baseline characteristics**			
Male sex	1067 (82%)	100 (92.6%)	0.005
Age (years)	38 (30–49)	49.5 (39.5–60)	< 0.001
Age group (years)			< 0.001
15–24	113 (8.7%)	3 (2.8%)	
25–34	391 (30.1%)	12 (11.1%)	
35–44	353 (27.1%)	24 (22.2%)	
45–54	250 (19.2%)	25 (23.1%)	
55–64	138 (10.6%)	28 (25.9%)	
≥ 65	56 (4.3%)	16 (14.8%)	
Nationality according to WHO region			0.005
African Region	27 (2.1%)	2 (1.9%)	
Eastern Mediterranean Region	449 (34.5%)	36 (33.3%)	
European Region	22 (1.7%)	2 (1.9%)	
Region of the Americas	11 (0.8%)	2 (1.9%)	
South-East Asia Region	739 (56.8%)	52 (48.1%)	
Western Pacific Region	53 (4.1%)	14 (13%)	
Healthcare workers	60 (4.6%)	3 (2.8%)	0.48
Pregnant	18 (1.4%)	1 (0.9%)	1.0
Diabetes mellitus	275 (21.1%)	52 (48.1%)	< 0.001
Hypertension	248 (19.1%)	52 (48.1%)	< 0.001
Coronary artery disease	31 (2.4%)	10 (9.3%)	< 0.001
Chronic lung disease	73 (5.6%)	10 (9.3%)	0.12
Chronic liver disease	14 (1.1%)	4 (3.7%)	0.043
Chronic kidney disease	21 (1.6%)	13 (12%)	< 0.001
Malignancy	18 (1.4%)	2 (1.9%)	0.66
Number of comorbidities			< 0.001
None	859 (66.0%)	35 (32.4%)	
One comorbidity	268 (20.6%)	33 (30.6%)	
Two comorbidities	124 (9.5%)	20 (18.5%)	
More than two comorbidities	50 (3.8%)	20 (18.5%)	
Current or past smoker	121/806 (15%)	9/52 (17.3%)	0.71
Mode of presentation			< 0.001
Screening or contact tracing	390/1293 (30.2%)	0	
Symptomatic	903/1293 (69.8%)	108 (100%)	
Symptoms			
Fever	710/1289 (55.1%)	105 (97.2%)	< 0.001
Cough	730/1289 (56.6%)	97 (89.8%)	< 0.001
Sore throat	345/1289 (26.8%)	21 (19.4%)	0.11
Rhinorrhea	130/1289 (10.1%)	1 (0.9%)	< 0.001
Dyspnea	156/1289 (12.1%)	56 (51.9%)	< 0.001
Fatigue	121/1289 (9.4%)	15 (13.9%)	0.13
Generalized pain	265/1289 (19.9%)	21 (19.4%)	0.9
Diarrhea	53/1289 (4.1%)	2 (1.9%)	0.43
Nausea and/or vomiting	52/1289 (4.0%)	10 (9.3%)	0.024
**Measurements, vital signs, and laboratory results within the first 24 h of hospitalization**			
Body mass index^a^ (kg/m^2^)	26.6 (23.8–29.7)	28.2 (25.8–31.6)	< 0.001
Systolic blood pressure^†^ (mmHg)	117.0 (108–127)	116 (103.5–126)	0.074
Temperature^b^ (^o^ C)	37.0 (36.8–37.9)	37.8 (37.1–38.7)	< 0.001
Hear rate^c^ (beats per minute)	86 (78–97)	95 (86–108)	< 0.001
Respiratory rate^d^ (breaths per minute)	19 (18–20)	26.5 (20–32.5)	< 0.001
Oxygen saturation^e^ (%)	98 (96–99)	94 (91–96)	< 0.001
White blood cell count^f^ (×10^9^ cells per L)	6.4 (5.0–7.9)	6.6 (5.2–8.6)	0.058
Lymphocyte count^g^ (× 10^9^ cells per L)	1.7 (1.2–2.2)	1.0 (0.7–1.3)	< 0.001
Platelet count^g^ (×10^9^ cells per L)	235 (189–283)	202.5 (171.5–242.5)	< 0.001
Serum sodium^h^ (mmol/L)	138 (136–140)	135 (133–137)	< 0.001
Serum creatinine^i^ (μmol/L)	80 (68–90)	90 (76.5–108.5)	< 0.001
CRP^j^ (mg/L)	7.0 (5–33.8)	107.7 (55.3–169.5)	< 0.001
ALT^k^ (U/L)	27 (19–40)	32 (21.5–50.5)	< 0.001
Chest radiology showing pulmonary infiltrates	457/1275 (35.8%)	102 (94.4%)	< 0.001
**Management**			
Highest respiratory support in the first 24 h of hospitalization			< 0.001
Ambient air	1226 (94.2%)	7 (6.5%)	
Oxygen via face mask or nasal canulae	75 (5.8%)	45 (41.7%)	
Non–invasive mechanical ventilation	0	14 (13%)	
Invasive mechanical ventilation	0	42 (38.9%)	
Invasive mechanical ventilation anytime during hospitalization	0	91 (84.3%)	< 0.001
Vasopressor support	2 (0.2%)	66 (61.1%)	< 0.001
Renal replacement therapy	4 (0.3%)	16 (14.8%)	< 0.001
Antiviral and Anti–inflammatory Therapy			
Hydroxychloroquine	936 (71.9%)	108 (100.0%)	< 0.001
Azithromycin	791 (60.8%)	107 (99.1%)	< 0.001
Lopinavir–ritonavir	435 (33.4%)	87 (80.6%)	< 0.001
Ribavirin	33 (2.5%)	74 (68.5%)	< 0.001
Interferon	1 (0.1%)	26 (24.1%)	< 0.001
Tocilizumab	12 (0.9%)	99 (91.7%)	< 0.001
Systemic corticosteroids	7 (0.5%)	71 (65.7%)	< 0.001
**Complications**			
Acute respiratory distress syndrome	0	94 (87%)	< 0.001
Acute kidney injury	20 (1.5%)	47 (43.5%)	< 0.001
Myocardial injury	4 (0.3%)	16 (14.8%)	< 0.001
Thromboembolism	0	3 (2.8%)	< 0.001
Arrhythmia	0	4 (3.7%)	< 0.001
**Length of stay (days)**			
Hospital length of stay	7 (3–12)	24.5 (19–37.5)	< 0.001
ICU length of stay	NA	12 (8–21)	NA

Data are median (IQR), n (%), or n/N (%), where N is the total number of patients with available data. *P* values comparing ICU and non-ICU are from Pearson’s chi-squared test, Fisher’s exact test, or Wilcoxon rank-sum test. ^a^Data missing for 246 (17.5%). ^b^Data missing for 16, (1.1%). ^c^Data missing for 179 (12.7%). ^d^Data missing for 33 (2.3%). ^e^Data missing for 20 (1.4%). ^f^Data missing for 37 (2.6%). ^g^Data missing for 42 (3%). ^h^Data missing for 39 (2.8%). ^i^Data missing for 43 (3.1%). ^j^Data missing for 148 (10.5%). ^k^Data missing for 83 (5.9%). *ALT* Alanine transaminase; *CRP* C-reactive protein; *ICU* Intensive care unit; *NA* Not applicable; *WHO* World Health Organization

### Risk factors for ICU admission

In univariable analysis, the odds of admission to ICU were significantly higher in older patients, males compared with females, and in those with diabetes, hypertension, coronary artery disease, or chronic lung, liver, or kidney disease, and in those with higher BMI (Table 2). The presence of cough, dyspnea, or fever, elevated baseline heart rate or respiratory rate, decreased oxygen saturation, lower lymphocyte count, and increased serum creatinine, CRP, and alanine transaminase (ALT) were also associated with admission to ICU (Table 2).

**Table 2:** Risk factors associated with admission of COVID-19 patients to ICU

Variable	Univariable OR (95% CI, ***P*** value)	Multivariable OR (95% CI, ***P*** value)
Age (per year increase)	1.056 (1.041–1.071, *P* < 0.001)	1.041 (1.022–1.061, *P* < 0.001)
Male sex (versus female)	2.741 (1.316–5.711, P 0.007)	4.375 (1.964–9.744, *P* < 0.001)
Co-morbidity present (versus not present)		
Diabetes mellitus	3.461 (2.319–5.164,*P* < 0.001)	1.698 (1.050–2.746, P 0.031)
Hypertension	2.919 (1.941–4.340, *P* < 0.001)	0.980 (0.589–1.630, P 0.937)
Coronary artery disease	4.180 (1.991–8.778, *P* < 0.001)	1.090 (0.449–2.643, P 0.849)
Chronic lung disease	1.717 (0.859–3.430, P 0.126)	..
Chronic liver disease	3.536 (1.143–10.934, P 0.028)	2.463 (0.716–8.465, P 0.153)
Chronic kidney disease	8.341 (4.050–17.177, *P* < 0.001)	3.590 (1.596–8.079, P 0.002)
BMI (per one kg/m^2^ increase)	1.067 (1.033–1.102, *P* < 0.001)	1.067 (1.027–1.108, P 0.001)
Presenting symptoms (versus not present)		
Fever	28.542 (9.011–90.401, *P* < 0.001)	
Cough	6.145 (3.339–11.312, *P* < 0.001)	
Dyspnea	7.879 (5.123–11.909, *P* < 0.001)	
Sore throat	0.660 (0.403–1.079, *P* < 0.001)	
Rhinorrhea	0.833 (0.0115–0.602, P 0.014)	
Baseline vital signa (per unit increase)		
Systolic blood pressure (mmHg)	0.987 (0.973–1.001, P 0.062)	
Heart rate (beats per minute)	1.037 (1.024–1.049, *P* < 0.001)	
Respiratory rate (breaths per minute)	1.264 (1.217–1.313, *P* < 0.001)	
Oxygen saturation (%)	0.651 (0.605–0.701, *P* < 0.001)	
Baseline laboratory results (per unit increase)		
Lymphocytes count (× 10^9^ per L)	0.155 (0.100–0.240, *P* < 0.001)	
Platelet count (× 10^9^ per L)	0.993 (0.990–0.996, *P* < 0.001)	
Serum sodium (mmol/L)	0.801 (0.757–0.848, *P* < 0.001)	
Serum creatinine (μmol/L)	1.003 (1.001–1.005, *P* < 0.005)	
CRP (mg/L)	1.016 (1.013–1.019, *P* < 0.001)	
ALT (U/L)	1.001 (0.999–1.002, P 0.2)	

*ALT* Alanine transaminase; *BMI* Body mass index (calculated as weight in kilograms divided by height in meters squared); *CI* Confidence interval; *COVID-19* Coronavirus disease 2019; *CRP* C-reactive protein; *ICU* Intensive care unit; *IQR* Interquartile range.; *OR* Odds ratio

In the multivariable logistic regression, we found that older age, male sex, co-existing diabetes or chronic kidney disease, and higher BMI were all independently associated with increased risk of need for ICU admission (Table 2).

### Fatal COVID-19

A total of 14 patients (0.28%) died within 60 days of follow up. The median age of fatal COVID-19 cases was 59.5 years (IQR 55.8–68). Most deceased patients were males (13, 92.9%) and most (8, 57.1%) had two or more co-morbidities (see Table S2 in Supplementary Material).

Two patients died without hospitalization. The first was a 59-year-old man with a history of hypertension and heavy smoking. He had asymptomatic SARS-CoV-2 infection and was isolated in a community facility pending viral clearance. He developed severe chest pain and cardiopulmonary arrest 17 days after COVID-19 diagnosis. The second patient was a 74-year-old man with end-stage kidney disease, hypertension, diabetes and coronary artery disease. He developed cardiopulmonary arrest shortly after presenting to the emergency department in severe respiratory distress. A post-mortem examination to confirm the cause of death was not performed in either case.

The remaining 12 deaths all occurred in patients who were in ICU with severe acute respiratory distress syndrome requiring prolonged invasive mechanical ventilation. Deaths occurred after a median of 24 days (IQR 14–49) from COVID-19 diagnosis. Ten (86.5%) deaths occurred in patients aged 55 or older. The remaining two were in patients aged 54 years and 24 years. The former had diabetes, hypertension, and obesity (BMI 38.7). The latter patient presented with fulminant hepatitis and his hepatitis B serology was positive for surface IgM antibodies. He died within 11 days with encephalopathy and multi-organ failure.

### Pregnant women with SARS-CoV-2 infection

The study included 26 pregnant women with SARS-CoV-2 infection with median age of 29 years (IQR 25.5–33). Nineteen (73.1%) were hospitalized, including one (3.8%) in ICU, and all were discharged within the follow up period. Ten (38.5%) pregnant women with COVID-19 gave birth during the follow up period; all resulting in healthy babies with negative SARS-CoV-2 tests (see Table S3 in Supplementary Material).

### Healthcare workers with SARS-CoV-2 infection

A total of 135 patients in this cohort were healthcare workers. Their median age was 35 years (28–43) and the majority were males (101, 74.8%). The most frequent professional background of affected healthcare workers was nursing (49, 36.3%), and allied healthcare (27, 20%) (see Table S4 in Supplementary Material). Out of 63 (46.6%) who required hospitalization, three (2.2%) required admission to ICU. All healthcare workers in this study were alive and out of hospital at end of follow up (see Table S4 in Supplementary Material).

### Children with SARS-CoV-2 infection

There were 131 individuals aged 14 years or less in the study, of which 69 (52.7%) were males. Median age was 7 years (IQR 4–10). Children were mostly diagnosed in the context of contact screening (75/123, 61%), and were not hospitalized (116, 88.5%). The majority (120, 91.6%) of children, including all seven infants, had family members with confirmed COVID-19 (see Table S5 in Supplementary Material).

## Discussion

In this national COVID-19 cohort, only 14 (0.28%) out of 5000 patients died within 60 days of diagnosis, and 12 (0.24%) required ongoing hospitalization at the end of the 60-day follow up period.

SARS-CoV-2 infection are generally slightly more common in males than females [14]. However Our report shows that 88.7% of SARS-CoV-2 infections in Qatar were in males. Our findings reflect the country’s demographic characteristics. Male to female ratio in Qatar’s general population is 2.8 and the corresponding male to female SARS-CoV-2 incidence per 100,000 population in our report is 2.1. Notably, the population’s male to female ratios are 3.5–3.7 in age groups between 25 to 54 (3.5–3.7), where 68.9% of SARS-CoV-2 infections where reported (Table S6, supplement).

Our mortality rates are considerably lower than previously reported form large COVID-19 cohorts from China, Europe and United States [5–9]. There are several possible explanations for our findings. Firstly, as of July 11, 2020, the total number of SARS-CoV-2 RT-PCR tests performed in Qatar is 142 per 1000 population, compared with 97.6 in Italy, 117.6 in the United States, and 102.8 in the United Kingdom [15]. Higher COVID-19-associated mortality rates are often reported from settings where COVID-19 testing is not readily available for those who do not have severe symptoms, thus skewing outcome assessments towards the severe end of the COVID-19’s clinical spectrum [2–4, 7]. On the other hand, nearly one third of patients reported in our study were identified through screening efforts. Our lower mortality rates could therefore be in part due to higher detection of milder COVID-19 cases.

Secondly, our cohort’s demographic profile is consistent with the country’s population being largely constituted of male migrants working in the country’s numerous infrastructure projects (Table S6, supplement). Older age and the presence of multiple co-morbidities have consistently been associated with increased risk of severe COVID-19, need for critical care support, and mortality [7, 9, 16]. The majority (83%) of patients in our study did not have any pre-existing chronic medical conditions. Moreover, with a median patient age of 35 years (IQR 28–43), our patients were considerably younger than those reported in large cohorts from Lombardy Region in Italy (median 63 year, IQR 56–70), the United Kingdom (median 73 years, IQR 58–82 years) and New York City (median 54 years, IQR 38–66) [5–7]. Note should also be taken of Qatar’s population being relatively younger than most countries reporting high COVID-19-associated mortality. For example, the median age in Qatar is only 33.2 years, whereas the median population age is 45.5 years in Italy, and 40.5 years in the United Kingdom [17]. In addition, the proportion of population aged over 65 years is only 1.3% in Qatar, while it is 23% in Italy and 18.5% in the United Kingdom [18].

A third factor in explaining our low COVID-19-associated mortality is the rapid escalation of the healthcare system’s capacity to accommodate the expected hike in demand for hospital beds in general, and for ICU support in particular. It has been suggested that some of the worst COVID-19-associated mortality rates have in part been the result of overwhelmed critical care resources that could not support a large influx of severely ill COVID-19 patients [3, 19]. This has stimulated discussions around rationing of critical care support for COVID-19 patients, including potentially difficult decisions to withdraw resources from one patient to provide them to another [20]. On the other hand, critical care support is rarely withheld in our setting, even in cases where the prognosis appears to be unfavorable.

While diabetes mellitus, coronary artery disease, chronic liver disease, hypertension and chronic kidney diseases all appeared to be associated with risk of admission to ICU in our univariate analysis, the association was statistically significant only for the latter wo in the adjusted logistic regression analysis (Table 2). This is probably the result of interactions between our cohort’s co-morbidities and their age.

Deaths observed in our study have largely occurred in older patients with multiple co-morbidities. Though 85.7% of deaths occurred in those aged 55 years or above, this group constituted only 7.4% of our entire cohort. Our age group-specific mortality was 2.5% in those aged 55–64 years, and 5.4% in those aged 65 years or more. These figures are comparable with mortality rates in similar age groups in China, Italy, and the United States, but are considerably lower than those reported from the United Kingdom [6, 8, 9, 21].

One patient in our cohort died while in a community isolation facility with asymptomatic SARS-CoV-2 infection. His rapid demise after complaining of chest pain suggests that his death was caused by myocardial infarction or pulmonary embolism. Both complications are increasingly recognized associations with COVID-19 [22, 23]. An increase in out-of-hospital cardiac arrests has been observed in association with SARS-CoV-2 pandemic, including in patients with symptoms compatible with COVID-19 [24]. Moreover, 14.8% of our ICU patients and 0.3% of our non-ICU patients had evidence of myocardial injury during their hospitalization. The diagnosis of COVID-19 in patients with known or increased risk of coronary artery disease should be an opportunity to review and optimize medical therapy to reduce the risk of acute coronary events.

Most hospitalized patients in our study received investigational antiviral therapies. However, recent reports from large cohort and randomized clinical trials do not support the use of hydroxychloroquine, alone or in combination with azithromycin, or lopinavir-ritonavir for patients with COVID-19 [25, 26]. It is likely that COVID-19 management will continue to evolve as more results from ongoing clinical trials become available [27].

Our analysis showed that increasing age, male sex, higher BMI, and the presence of diabetes or chronic kidney disease are risk factors for admission to ICU. Remarkably, hypertension, chronic lung disease, and coronary artery disease, all of which are frequently reported as important predictors for severe COVID-19 in previous studies, were not independently associated with ICU admission in our setting [28]. Furthermore, our univariable analysis showed that presenting with dyspnea and cough as well as baseline blood abnormalities such as lower lymphocyte count, higher CRP and serum creatinine are associated with increased risk of admission to ICU [28, 29].

Higher BMI as a risk factor for severe COVID-19 is particularly noteworthy [30]. Median BMI in our hospitalized patients was 26.8 kg/m^2^ (IQR 23.9–29.8), a reflection of the growing concern over the increasing prevalence of overweight and obesity in developing countries, along with its consequent health problems such as diabetes and cardiovascular disease [31]. In the context of COVID-19, it is important to recognize the role of overweight and obesity as a driver of severe COVID-19. Our findings help guide deployment of medical resources to better select patients for hospitalization, closer clinical monitoring, and early clinical support.

Healthcare workers represented 2.7% of cases in our report. Three (2.2%) of our healthcare workers required admission to ICU. Unlike some unfortunate reports from elsewhere, all healthcare workers in our study fully recovered within the study follow up period [32]. Risk to healthcare personnel is highest in those with prolonged direct contact with symptomatic patients, especially where personal protective equipment are either in short supply or not used appropriately [33]. Also noteworthy is that 28.2% of healthcare workers in this study were asymptomatic. Single center healthcare worker screening studies reported asymptomatic rates ranging from 12.2 to 34% [34, 35]. The most efficient healthcare worker screening strategy that combines practicality with patient protection is still unclear.

Like previous reports, children in our study had a largely uneventful SARS-CoV-2 infection [36].

While only 28.5% of the entire cohort were hospitalized, the majority (73.1%) of pregnant women with COVID-19 in our report were hospitalized. However, only one (3.8%) out of 26 pregnant women in this report required admission to ICU, and none died within 60 days of follow up. Our findings are consistent with recent reports indicating that pregnancy may be independently associated with increased risk of hospitalization and severe COVID-19 [37, 38].

The limitations of this study include its observational nature and missing data for some variables. To address those limitations, we used multivariate analyses with multiple imputations to assess independent associations with the outcome. Despite this, our study benefits from being, to the best of our knowledge, the first to report 60-day outcomes of SARS-CoV-2, and to do so at a nationwide level.

## Conclusions

In a setting of proactive SARS-CoV-2 case finding, a younger population, and low co-morbidity burden, SARS-CoV-2 was associated with low all-cause mortality. Independent risk factors for ICU admission included older age, male sex, higher BMI, and co-existing diabetes or chronic kidney disease.

## Supplementary information

**Additional file 1 Table S1**. Baseline characteristics and outcomes of 5000 individuals with Coronavirus Disease 2019 in Qatar. **Table S2**. Coronavirus Disease 2019-associated deaths in Qatar. **Table S3**. Pregnant women with Coronavirus Disease 2019 in Qatar. **Table S4**. Healthcare Workers with Coronavirus Disease 2019 in Qatar. **Table S5**. Children with Coronavirus Disease 2019 in Qatar. **Table S6**. Qatar population and corresponding SARS-CoV-2 infection incidence per 100,000 population by sex and age group.

## Data Availability

The datasets used and analyzed during the current study are available from the corresponding author on reasonable request.
