# Supplementary material for: The first consecutive 5000 patients with Coronavirus Disease 2019 from Qatar; a nation-wide cohort study
Source: BMC Infect Dis. 2020 Oct 19;20:777. doi: 10.1186/s12879-020-05511-8 (PMC7570422; doi:10.1186/s12879-020-05511-8)
Supplement: Supplementary file 1 — Additional file 1 Table S1. Baseline characteristics and outcomes of 5000 individuals with Coronavirus Disease 2019 in Qatar. Table S2. Coronavirus Disease 2019-associated deaths in Qatar. Table S3. Pregnant women with Coronavirus Disease 2019 in Qatar. Table S4. Healthcare Workers with Coronavirus Disease 2019 in Qatar. Table S5. Children with Coronavirus Disease 2019 in Qatar. Table S6. Qatar population and corresponding SARS-CoV-2 infection incidence per 100,000 population by sex and age group. [file 12879_2020_5511_MOESM1_ESM.docx]

**Supplementary Data**

The First Consecutive 5000 Patients with COVID-19 in Qatar; a Nation-wide Cohort Study

Ali S. Omrani ^1,^*, Muna A. Almaslamani ^2^, Joanne Daghfal ^3^, Rand A. Alattar ^4^, Mohamed Elgara ^5^, Shahd H. Shaar ^6^, Tawheeda B. H. Ibrahim ^7^, Ahmed Zaqout ^8^, Dana Bakdach ^9^, Abdelrauof M. Akkari ^10^, Anas Baiou ^11^, Bassem Alhariri ^12^, Reem Elajez ^13^, Ahmed A. M. Husain ^14^, Mohamed N. Badawi ^15^, Fatma Ben Abid ^16^, Sulieman Abu Jarir ^17^, Shiema Abdalla ^18^, Anvar Kaleeckal ^19^, Kris Choda ^20^, Venkateswara R. Chinta ^21^, Mohamed A. Sherbash ^22^, Khalil Al-Ismail ^23^, Mohammed Abukhattab ^24^, Ali Ait Hssain ^25^, Peter V. Coyle ^26^, Roberto Bertollini ^27^, Michael P. Frenneaux ^28^, Abdullatif Al Khal ^29^, and Hanan M. Alkawari ^30^

^1^ Communicable Diseases Center, Hamad Medical Corporation, Doha, Qatar; aomrani@hamad.qa

^2^ Communicable Diseases Center, Hamad Medical Corporation, Doha, Qatar; malmaslamani@hamad.qa

^3^ Communicable Diseases Center, Hamad Medical Corporation, Doha, Qatar; jnader@hamad.qa

^4^ Communicable Diseases Center, Hamad Medical Corporation, Doha, Qatar; ralattar@hamad.qa

^5^ Medical Residency Program, Hamad Medical Corporation, Doha, Qatar; melgara@hamad.qa

^6^ Communicable Diseases Center, Hamad Medical Corporation, Doha, Qatar; sshaar@hamad.qa

^7^ Communicable Diseases Center, Hamad Medical Corporation, Doha, Qatar; tibrahim3@hamad.qa

^8^ Communicable Diseases Center, Hamad Medical Corporation, Doha, Qatar; azaqout@hamad.qa

^9^ Division of Critical Care Medicine, Hamad Medical Corporation, Doha, Qatar; dbakdach@hamad.qa

^10^ Division of Critical Care Medicine, Hamad Medical Corporation, Doha, Qatar; aakkari@hamad.qa

^11^ Division of Critical Care Medicine, Hamad Medical Corporation, Doha, Qatar; abaiou@hamad.qa

^12^ Hazm Mebaireek General Hospital, Hamad Medical Corporation, Doha, Qatar; balhariri@hamad.qa

^13^ Hamad General Hospital, Hamad Medical Corporation, Doha, Qatar; relajez@hamad.qa

^14^ Communicable Diseases Center, Hamad Medical Corporation, Doha, Qatar; ahusain@hamad.qa

^15^ Communicable Diseases Center, Hamad Medical Corporation, Doha, Qatar; mmohamed21@hamad.qa

^16^ Communicable Diseases Center, Hamad Medical Corporation, Doha, Qatar; fabid@hamad.qa

^17^ Communicable Diseases Center, Hamad Medical Corporation, Doha, Qatar; sabujarir@hamad.qa

^18^ Communicable Diseases Center, Hamad Medical Corporation, Doha, Qatar; sahmed84@hamad.qa

^19^ Business Intelligence Unit, Hamad Medical Corporation, Doha, Qatar; akaleeckal@hamad.qa

^20^ Business Intelligence Unit, Hamad Medical Corporation, Doha, Qatar; kchoda@hamad.qa

^21^ Business Intelligence Unit, Hamad Medical Corporation, Doha, Qatar; vchinta@hamad.qa

^22^ Rumailah Hospital, Hamad Medical Corporation, Doha, Qatar; msherbash@hamad.qa

^23^ Communicable Diseases Center, Hamad Medical Corporation, Doha, Qatar; kismail4@hamad.qa

^24^ Communicable Diseases Center, Hamad Medical Corporation, Doha, Qatar; mabukhattab@hamad.qa

^25^ Division of Critical Care Medicine, Hamad Medical Corporation, Doha, Qatar; ahssain@hamad.qa

^26^ Division of Virology, Hamad Medical Corporation, Doha, Qatar; pcoyle@hamad.qa

^27^ Ministry of Public Health, Doha, Qatar; rbertollini@moph.gov.qa

^28^ Academic and Faculty Affairs, Hamad Medical Corporation, Doha, Qatar; mfrenneaux@hamad.qa

^29^ Communicable Diseases Center, Hamad Medical Corporation, Doha, Qatar; aalkhal@hamad.qa

^30^ Ministry of Public Health, Doha, Qatar; hkuwari@moph.gov.qa

***** Correspondence: aomrani@hamad.qa; Tel.: +974 4025 4065 (Qatar)

**Table S1. Baseline characteristics and outcomes of 5000 individuals with Coronavirus Disease 2019 in Qatar**

| **Characteristic** | **Total cohort**  **(n = 5000)** | **Hospitalized patients**  **(n = 1424)** | **Non-hospitalized patients**  **(n = 3576)** | **P value** |
| --- | --- | --- | --- | --- |
| Male gender | 4436 (88.7%) | 1174 (82.4%) | 3262 (91.2%) | <0.001 |
| Age (years) | 35 (28-43) | 39 (30-50) | 33 (27-40) | <0.001 |
| Age group (years) | | | | <0.001 |
| ≤14 | 131 (2.6%) | 15 (1.1%) | 116 (3.2%) |  |
| 15–24 | 540 (10.8%) | 116 (8.1%) | 424 (11.9%) |  |
| 25–34 | 1811 (36.2%) | 403 (28.3%) | 1408 (39.4%) |  |
| 35–44 | 1445 (28.9%) | 377 (26.5%) | 1068 (29.9%) |  |
| 45–54 | 704 (14.1%) | 275 (19.3%) | 429 (12.0%) |  |
| 55–64 | 277 (5.5%) | 166 (11.7%) | 111 (3.1%) |  |
| ≥65 | 92 (1.8%) | 72 (5.1%) | 20 (0.6%) |  |
| Nationality according to WHO region | | | | <0.001 |
| African region | 164 (3.3%) | 29 (2%) | 135 (3.8%) |  |
| Eastern Mediterranean Region | 1293 (25.9%) | 497 (34.9%) | 796 (22.3%) |  |
| European Region | 46 ( 0.9%) | 24 (1.7%) | 22 (0.6%) |  |
| Region of the Americas | 24 ( 0.5%) | 13 (0.9%) | 11 (0.3%) |  |
| South-East Asia Region | 3311 (66.2%) | 794 (55.8%) | 2517 (70.4%) |  |
| Western Pacific Region | 162 ( 3.2%) | 67 (4.7%) | 95 (2.7%) |  |
| Healthcare workers | 135 (2.7%) | 63 (4.4%) | 72 (2%) | <0.001 |
| Pregnant | 26 (0.5%) | 19 (1.3%) | 7 (0.2%) | <0.001 |
| Diabetes mellitus | 470 (9.4%) | 327 (23%) | 143 (4%) | <0.001 |
| Hypertension | 476 (9.5%) | 292 (20.5%) | 184 (5.1%) | <0.001 |
| Coronary artery disease | 61 (1.2%) | 41 (2.9%) | 20 (0.6%) | <0.001 |
| Chronic lung disease | 156 (3.1%) | 87 (6.1%) | 69 (1.9%) | <0.001 |
| Chronic liver disease | 20 (0.4%) | 18 (1.3%) | 2 (0.1%) | <0.001 |
| Chronic kidney disease | 44 (0.8%) | 34 (2.4%) | 8 (0.2%) | <0.001 |
| Malignancy | 31 (0.6%) | 20 (1.4%) | 11 (0.3%) | <0.001 |
| Number of comorbidities | | | | <0.001 |
| None | 4151 (83.0%) | 905 (63.6%) | 3246 (90.8%) |  |
| One | 548 (11.0%) | 305 (21.4%) | 243 (6.8%) |  |
| Two | 215 ( 4.3%) | 144 (10.1%) | 71 (2%) |  |
| More than two | 86 ( 1.7%) | 70 (4.9%) | 16 (0.4%) |  |
| 60-day all-cause mortality | 14 (0.28%) | 12 (0.8%) | 2 (0.1%) | <0.001 |

Data are median (IQR) or number (%).WHO, World Health Organization

**Table S2. Coronavirus Disease 2019-associated deaths in Qatar**

| **Characteristic** | **Total (n = 14)** |
| --- | --- |
| Male gender | 13 (92.9%) |
| Nationality according to WHO region | |
| Eastern Mediterranean Region | 7 (50.0%) |
| South-East Asia Region | 6 (42.9%) |
| Region of the Americas | 1 (7.1%) |
| Age (years) | 59.5 (55.8–68) |
| Age group (years) | |
| ≤14 | 0 |
| 15–24 | 1 (7.1%) |
| 25–34 | 0 |
| 35–44 | 0 |
| 45–54 | 1 (7.1%) |
| 55–64 | 7 (50%) |
| ≥65 | 5 (35.7%) |
| **Co-morbidities** | |
| Diabetes mellitus | 10 (71.4%) |
| Hypertension | 6 (42.9%) |
| Coronary artery disease | 2 (14.3%) |
| Chronic lung disease | 0 |
| Chronic liver disease | 2 (14.3%) |
| Chronic kidney disease | 6 (42.9%) |
| Number of comorbidities | |
| None | 2 (14.3%) |
| One comorbidity | 4 (28.6%) |
| Two comorbidities | 2 (14.3%) |
| More than two comorbidities | 6 (42.9%) |
| Disposition | |
| Not hospitalized | 2 (14.3%) |
| Hospitalized in ICU | 12 (85.7%) |
| Complications | |
| Severe ARDS | 12 (85.7%) |
| Acute kidney injury requiring dialysis | 9 (64.3%) |
| Septic shock | 5 (35.7%) |
| Multi-organ failure | 8 (57.1%) |
| Time to death (days from diagnosis) | 24 (14–49) |

Data are n (%) or median (IQR). ARDS, acute respiratory distress syndrome; COVID-19, Coronavirus Disease 2019; ICU, intensive care unit; WHO, World Health Organization

**Table S3. Pregnant women with Coronavirus Disease 2019 in Qatar**

| **Characteristic** | **Total (n = 26)** |
| --- | --- |
| Age (years) | 29 (25.5–33) |
| **Nationality according to WHO region** | |
| Eastern Mediterranean Region | 20 (76.9%) |
| European Region | 1 (3.8%) |
| South-East Asia Region | 4 (15.4%) |
| Western Pacific Region | 1 (3.8%) |
| Healthcare workers | 2 (7.7%) |
| **Comorbidities** | |
| Diabetes mellitus | 2 (7.7%) |
| Chronic lung disease | 3 (11.5%) |
| **Mode of presentation** | |
| Screening or contact tracing | 8 (30.8%) |
| Symptomatic | 18 (69.2%) |
| **COVID severity** | |
| Mild/asymptomatic | 22 (84.6%) |
| Moderate | 1 (3.8%) |
| Severe | 2 (7.7%) |
| Critical | 1 (3.8%) |
| **Disposition** | |
| Not hospitalized | 7 (26.90%) |
| Hospitalized, non-ICU | 18 (69.2%) |
| Hospitalized, ICU | 1 (3.8%) |
| **60 day Outcomes** | |
| Died | 0 |
| Still in hospital | 0 |
| Still in ICU | 0 |
| Gestational age | 25 (19–32) |
| **Trimester** |  |
| First | 3 (11.5%) |
| Second | 10 (38.5%) |
| Third | 13 50%) |
| **Pregnancy outcome** | |
| Abortion/miscarriage | 2 (7.7%) |
| Live birth | 10 (38.5%) |
| Still pregnant | 14 (53.8%) |

Data are n (%) or median (IQR). COVID-19, Coronavirus Disease 2019; ICU, intensive care unit; WHO, World Health Organization

**Table S4. Healthcare Workers with Coronavirus Disease 2019 in Qatar**

| **Characteristic** | **Total (n = 135)** |
| --- | --- |
| Male gender | 101 (74.8%) |
| **Nationality according to WHO region** | |
| Eastern Mediterranean Region | 42 (31.1%) |
| European Region | 4 (3%) |
| South-East Asia Region | 74 (54.8%) |
| Western Pacific Region | 15 (11.1%) |
| Age (years) | 35 (28–43) |
| **Age group (years)** | |
| 15–24 | 42 (31.1%) |
| 25–34 | 48 (35.6%) |
| 35–44 | 44 (32.6%) |
| 45–54 | 32 (23.7%) |
| 55–64 | 6 (4.4%) |
| ≥65 | 1 (0.7%) |
| **Type of healthcare job** | |
| Nursing | 49 (36.3%) |
| Allied Health | 27 (20.0%) |
| Service/Support/Maintenance | 18 (13.3%) |
| Administration | 14 (10.4%) |
| Physician | 10 (7.4%) |
| Ambulance service | 9 (6.7%) |
| Laboratory staff | 8 (5.9%) |
| **Comorbidities** | |
| Diabetes mellitus | 16 (11.9%) |
| Hypertension | 27 (20%) |
| Coronary artery disease | 3 (2.2%) |
| Chronic lung disease | 13 (9.6%) |
| Chronic kidney disease | 1 (0.7%) |
| **Presentation** | |
| Screening | 37/131 (28.2%) |
| Symptomatic | 94/131 (71.8%) |
| **COVID severity** | |
| Mild/asymptomatic | 109 (80.7%) |
| Moderate | 22 (16.3%) |
| Severe | 2 (1.5%) |
| Critical | 2 (1.5%) |
| **Disposition** | |
| Not hospitalized | 72 (53.3%) |
| Hospitalized, non-ICU | 60 (44.4%) |
| Hospitalized, ICU | 3 (2.2%) |
| **60 day outcomes** | |
| Died | 0 |
| Still in hospital | 0 |
| Still in ICU | 0 |

Data are median (IQR), n (%), or n/N (%), where N is the total number of patients with available data. COVID-19, Coronavirus Disease 2019; ICU, intensive care unit; WHO, World Health Organization

**Table S5. Children with Coronavirus Disease 2019 in Qatar**

| **Characteristic** | **Total (n = 131)** |
| --- | --- |
| Male gender | 69 (52.7%) |
| Age (years) | 7 (4–10) |
| **Nationality according to WHO region** |  |
| Eastern Mediterranean Region | 105 (80.2%) |
| European Region | 3 (2.3%) |
| Region of the Americas | 2 (1.5%) |
| South-East Asia Region | 20 (15.3%) |
| Western Pacific Region | 1 (0.8%) |
| **Presentation** |  |
| Screening | 75/123 (61%) |
| Symptomatic | 48/123 (39%) |
| **Disposition** |  |
| Not hospitalized | 116 (88.5%) |
| Hospitalized, non-ICU | 15 (11.5%) |
| Family contacts with confirmed SARS-CoV-2 infection | 120 (91.6%) |
| **60 day outcomes** |  |
| Died | 0 |
| Still in hospital | 0 |
| Still in ICU | not applicable |

Data are median (IQR), n (%), or n/N (%), where N is the total number of patients with available data. COVID-19, Coronavirus Disease 2019; ICU, intensive care unit; WHO, World Health Organization

**Table S6. Qatar population and corresponding SARS-CoV-2 infection incidence per 100,000 population by sex and age group.**

| **Age group (years)** | **Total Population in Qatar** | **Incidence for total population (per 100,000)** | **Male population in Qatar** | **Incidence for males (per 100,000)** | **Female population in Qatar** | **Incidence for females (per 100,000)** | **Male to Female ratio in population** | **Male to Female ratio in incidence per 100,000 population** |
| --- | --- | --- | --- | --- | --- | --- | --- | --- |
| ≤14 | 398,435 (14.2%) | 32.9 | 203,153 | 34.0 | 195,282 | 31.8 | 1.0 | 1.1 |
| 15–24 | 336,454 (12.0%) | 160.5 | 253,759 | 182.9 | 82,695 | 90.7 | 3.1 | 2.0 |
| 25–34 | 948,210 (33.9%) | 191.0 | 746,260 | 218.0 | 201,950 | 91.1 | 3.7 | 2.4 |
| 35–44 | 682,609 (24.4%) | 211.7 | 531,085 | 251.6 | 151,524 | 71.9 | 3.5 | 3.5 |
| 45–54 | 296,506 (10.6%) | 237.4 | 231,631 | 275.0 | 64,875 | 103.3 | 3.6 | 2.7 |
| 55–64 | 104,613 (3.7%) | 264.8 | 78,208 | 304.3 | 26,405 | 151.5 | 3.0 | 2.0 |
| ≥65 | 32,375 (1.2%) | 284.2 | 20,180 | 322.1 | 12,195 | 221.4 | 1.7 | 1.5 |
| Total | 2,799,202 |  | 2,064,276 | 158.5 | 734,926 | 76.7 | 2.8 | 2.1 |
